# Comparing fiberoptic bronchoscopy- and a tracheal tube-mounted camera-guided percutaneous dilatational tracheostomy

**DOI:** 10.1186/s13054-018-1960-x

**Published:** 2018-02-07

**Authors:** Fu-Shan Xue, Chao Wen, Ya-Yang Liu

**Affiliations:** Department of Anesthesiology, Plastic Surgery Hospital, Chinese Academy of Medical Sciences and Peking Union Medical College, 33 Ba-Da-Chu Road, Shi-Jing-Shan District, Beijing, 100144 People’s Republic of China

In a small, prospective, randomized trial comparing fiberoptic bronchoscopy (FOB) and an endotracheal tube-mounted camera (VivaSight TM-SL tube [VST]; ETView, Misgav, Israel) for optical guidance of percutaneous dilatational tracheostomy (PDT), Grensemann et al. [1] concluded that visualization of PDT with the VST is not non-inferior to the FOB, but ventilation is superior with less hypercarbia when using the VST. We note that in the FOB group, a thick FOB with an outer diameter of 4.9 mm (Olympus BF-P60; Olympus Medical Systems Corp., Tokyo, Japan) was used. The readers were not provided with the sizes of endotracheal tubes used in the FOB group, but placement of such a thick FOB into an adult endotracheal tube can significantly impair ventilation, especially for critically ill patients requiring PDT [2]. This may be a main reason for the inferior ventilation with more hypercarbia using FOB in this study. We argue that different results would have been obtained if a thin adult FOB, such as Olympus BF-DP with an outer diameter of 3.1 mm, was used. Furthermore, it was unclear whether a comparable fresh gas flow was used for mechanical ventilation during the PDT procedure. This could confuse the interpretation of more hypercarbia with the FOB.

For critically ill patients, the time required for the PDT procedure is the main concern. In the *Methods* section, Grensemann et al. did not clearly define the procedure duration. In the *Results* section, the authors reported that the mean procedure duration from skin incision to insertion of tracheal cannula did not differ significantly between groups. This may not be an appropriate comparison. We believe that the total procedure duration of PDT should be the time taken from insertion of direct laryngoscopy for tube exchange to completion of PDT in the VST group and from insertion of FOB to completion of PDT in the FOB group.

Finally, other than airway visualization, during the PDT procedure, the versatility of FOB can also offer invaluable advantages, such as determination of incision site, avoidance of accidental extubation, detection and management of complications, etc. [3, 4]. The recent evidence indicates that PDT combined with FOB not only is a time-saving, easy-to-operate technique with few complications, but can manage complications of posterior tracheal wall injury or perforation, tracheoesophageal fistula [5]. In the absence of high-quality evidence establishing the safety of the VST-guided PDT procedure, we believe that FOB is still the most frequently used technique for monitoring the PDT procedure while maintaining mechanical ventilation.

## Abbreviations

FOB: Fiberoptic bronchoscopy
PDT: Percutaneous dilatational tracheostomy
VST: VivaSight TM-SL tube
